# Creep-recovery behaviors of articular cartilage under uniaxial and biaxial tensile loadings

**DOI:** 10.3389/fbioe.2022.1085062

**Published:** 2023-01-10

**Authors:** Lilan Gao, Gang Liu, Yansong Tan, Ruixin Li, Chunqiu Zhang, Hong Gao, Bingjie Zhao

**Affiliations:** ^1^ Tianjin Key Laboratory for Advanced Mechatronic System Design and Intelligent Control, School of Mechanical Engineering, Tianjin University of Technology, Tianjin, China; ^2^ National Demonstration Center for Experimental Mechanical and Electrical Engineering Education, Tianjin University of Technology, Tianjin, China; ^3^ Tianjin Stomatological Hospital, The Affiliated Stomatological Hospital of Nankai University, Tianjin Key Laboratory of Oral and Maxillofacial Function Reconstruction, Tianjin, China; ^4^ School of Chemical Engineering and Technology, Tianjin University, Tianjin, China

**Keywords:** articular cartilage, biaxial loading, stress ratio, creep-recovery, strain ratio

## Abstract

Creep deformation in cartilage can be observed under physiological loads in daily activities such as standing, single-leg lunge, the stance phase of gait. If not fully recovered in time, it may induce irreversible damage in cartilage and further lead to early osteoarthritis. In this study, 36 cruciform-shape samples in total from 18 bulls were employed to conduct the uniaxial and biaxial creep-recovery tests by using a biaxial cyclic testing system. Effects of stress level (*σ* = .5, 1.0, 1.5 MPa) and biaxial stress ratio (*B* = 0, .3, .5, 1.0) on creep-recovery behaviors of cartilage were characterized. And then, a viscoelastic constitutive model was employed to predict its creep-recovery behaviors. The results showed that the creep strain and its three components, namely instantaneous elastic strain, delayed elastic strain and viscous flow strain, increase with the increasing stress level or with the decreasing biaxial stress ratio. Compared with uniaxial creep-recovery, biaxial creep-recovery exhibits a smaller creep strain, a faster recovery rate of creep strain and a smaller residual strain. Besides, the built viscoelastic model can be used to describe the uniaxial creep-recovery behaviors of cartilage as a good correlation between the fitted results and test results is achieved. The findings are expected to provide new insights into understanding normal joint function and cartilage pathology.

## Graphical Abstract

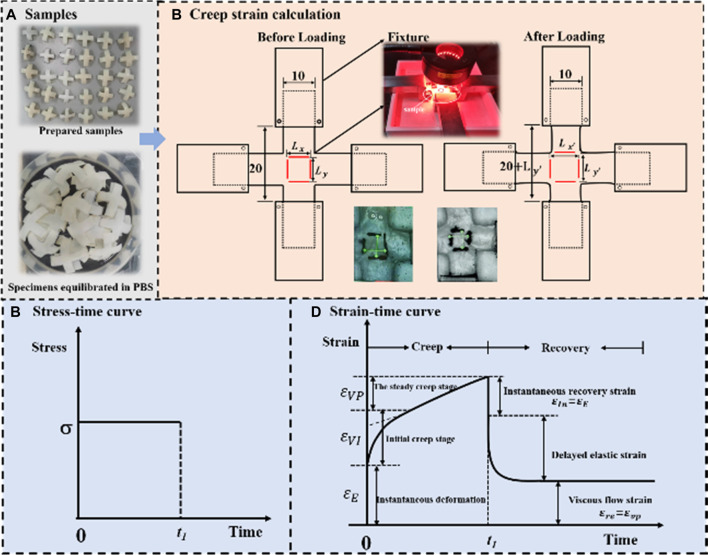

## Highlights

• Creep-recovery behaviors are explored at different stress levels and biaxial stress ratios.
• Creep strain and its components increase with stress level.
• A biaxial stress ratio of 1 imposes the smallest creep strain.
• Increasing biaxial stress ratio accelerates the recovery of creep strain and decreases the residual strain.
• The higher the biaxial stress ratio, the smaller the viscous flow strain and the delayed elastic strain.

## 1 Introduction

Articular cartilage has the functions to absorb shocks, transmit loads and sustain complex mechanical loading histories, thus maintaining dynamic mechanical equilibrium of knee joint in daily activities. As a kind of fluid saturated porous material, articular cartilage exhibits viscoelastic responses under mechanical loads. And creep is observed in cartilage when the mechanical load is held constant during standing, single-leg lunge (Hosseini et al., 2010; Choi et al., 2016; Qiu et al., 2016), the stance phase of gait (Liu et al., 2010; Li et al., 2014) and so on. Creep deformation under physiological loads may serve as a functional indicator for cartilage healthy. The majority of creep deformation in cartilage can recover timely. However, long term overloading may cause irreversible deformation, further induce damage in cartilage and lead to early osteoarthritis (Roos et al., 1998; Felson, 2013; Martel-Pelletier et al., 2016; Georgiev and Angelov, 2019; Peters et al., 2022 **)**. Studying the creep and creep-recovery behaviors of articular cartilage are helpful for understanding the normal joint function and cartilage pathology.

*In vivo* creep tests were usually conducted by applying 50%–150% body weight on the joint for 300–900 s (Herberhold et al., 1998; Herberhold et al., 1999; Hosseini et al., 2010; Choi et al., 2016; Uzuner et al., 2020), and the creep deformations of cartilage were measured *in vivo* by using imaging techniques like computed tomography (CT), magnetic resonance (MR), dual fluoroscopic imaging (DF). Herberhold et al. found that the creep deformation had high inter-individual variability (Herberhold et al., 1998; 1999). Hosseini et al. found the cartilage deformation of human tibiofemoral increases sharply in the first 20 s and almost held constant beyond 50 s when a constant full body weight was quickly applied and maintained for 300 s (Hosseini et al., 2010). Choi et al. studied the time-dependent creep behavior of tibial cartilage by applying a load of 75% body weight on limb and they reported that the creep deformation increased rapidly over the first minute and kept stable after 5 min (Choi et al., 2016). Uzuner et al. reported a substantial increase in cartilage deformation when joint loading increased from nil to 75% body weight and a continued small increase over time when the joint loading was held constant (Uzuner et al., 2020). *In vivo* experimental data help to understand the deformation characters of cartilage under physiological loads. However, technical limitations impede the accurate measurement of creep recovery in the physiological range and it non-etheless has significant difficulty to quantitatively analyze the creep-recovery deformation law of cartilage.

To cover the shortage of *in vivo* tests, *in vitro* creep tests are widely used to investigate the creep and creep-recovery behaviors of cartilage. Athanasiou et al. and Stolberg et al. employed the indentation test system to characterize the creep-recovery behavior of cartilage and they found that the initial creep rate is larger than the initial recovery rate and more than 95% of the creep deformation could recovered within 90 min (Athanasiou et al., 1991; Stolberg-Stolberg et al., 2018). Boettcher et al. found that the cartilage deformation increased with the increasing stress. (Boettcher et al., 2016). Cutclife and DeFrate validated the use of the recovery response for mechanical characterization of cartilage in a controlled, *ex vivo* environment **(** Cutcliffe and DeFrate, 2020). Reuter and Hurschler used a biphasic 3D-FE-based method to determine the biomechanical properties of equine articular cartilage (Reuter and Hurschler., 2021). In our previous study, the depth-dependent creep strain of cartilage under uniaxial compressive load was investigated by an optimized digital image correlation (DIC) technique (Si et al., 2022). And the creep deformation could accelerate the fatigue damage of cartilage in knee joint (Gao et al., 2019). It should be mentioned that these studies on the creep-recovery behaviors of articular cartilage were probed under uniaxial loading.

The cartilage with freely squeezing and stretching *in vivo* is subjected to biaxial or even multiaxial loads, and however literature provides limited data on these. Thus, it is of great significance to study the biaxial or multiaxial creep-recovery behaviors of cartilage. The goal of this study is to investigate the creep-recovery behaviors of articular cartilage under biaxial tensile load. The hypothesize is that changing the stress state from uniaxial loading to biaxial loading has a significant effect on creep deformation of cartilage. To verify the hypothesize, both the uniaxial and biaxial creep-recovery experiments were conducted by using a biaxial cyclic testing system. Effects of stress level and biaxial stress ratio on cartilage creep-recovery were characterized. Creep strain components were analyzed and compared at different loading conditions. Additionally, the creep-recovery behaviors of cartilage were predicted by mathematical models. This study is original because it provides the first insight into the effect of biaxial constraint on tensile creep behavior of cartilage. The results could help to explore intrinsic mechanical properties of articular cartilage and to prevent cartilage damage.

## 2 Materials and methods

### 2.1 Sample preparation

**FIGURE 1:**
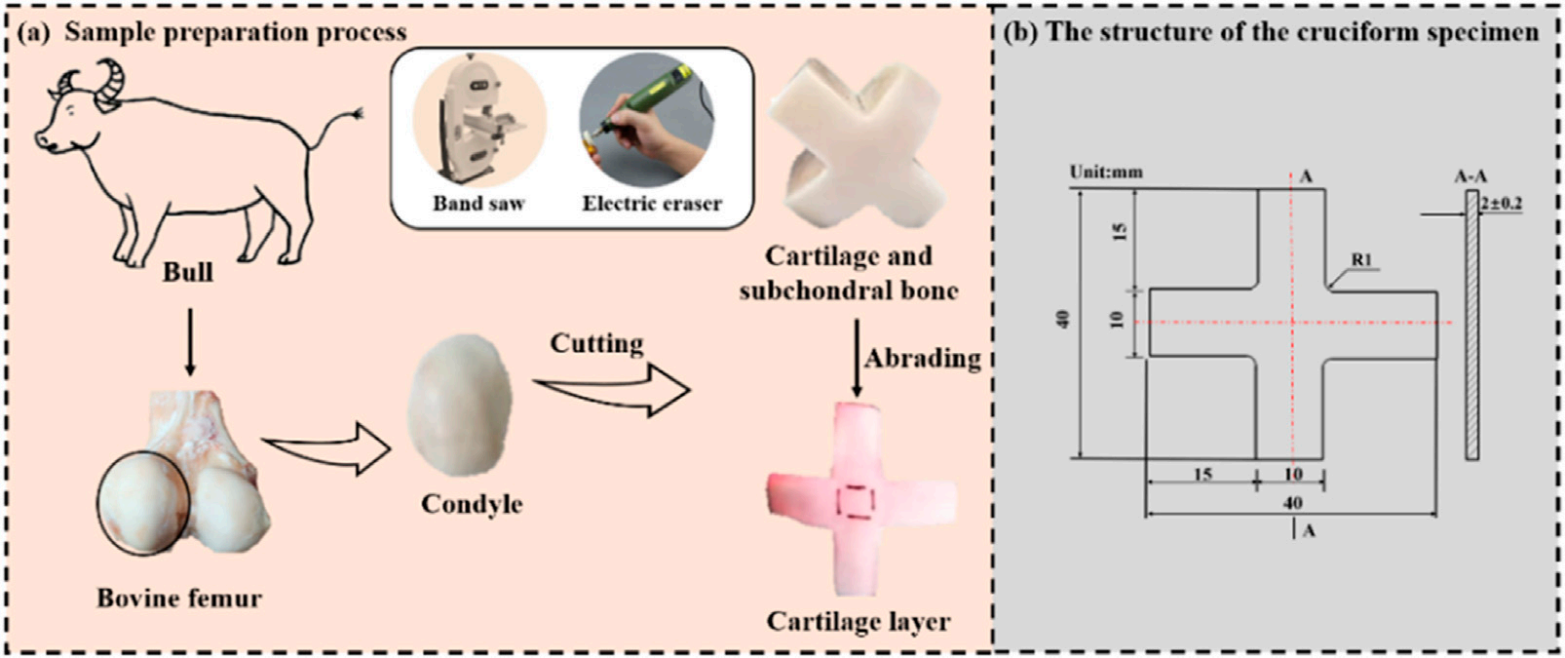
**(A)** Sample preparation process; **(B)** the structure of the cruciform specimen.

In total, 36 cruciform-shape samples from 18 bulls (around 8 months) were prepared for mechanical tests. The cruciform-shape samples were selected as test samples since they were widely used in biaxial tests to measure in plane mechanical properties of anisotropic material (Leotoing et al., 2013; Lamkanfi et al., 2015; Chen et al., 2022) and the sample preparation process in this study is similar to literature (Kamalanathan and Broom, 1993). As shown in Figure 1A , firstly, the cartilage with subchondral bone was harvested from the medial and lateral femoral trochleae of each femur. Secondly, the cruciform shape was marked on the cartilage with subchondral bone. Thirdly, the cartilage with subchondral bone was cut into the cruciform shape by a band saw. Finally, the subchondral bone was removed from cartilage by cutting with the band saw and abrading with an electric eraser (Si et al., 2022). Thus, the cruciform-shape cartilage sample with the thickness of 2.00 mm ± .2 mm was obtained. Figure 1B shows the specific structural dimensions of the cruciform specimen. It should be mentioned that since the curvature structure of cartilage induced by the orientation of collagen fibrils may affect the results, all the samples were cut from the same position of the femur. The pre-prepared samples were wrapped in gauze moistened with phosphate buffered saline and placed in a refrigerator at −20°C to preserve the collagen fibers intact of cartilage samples. Prior to tests, the samples were defrosted by being immersed in phosphate Buffered Saline (PBS, pH 7.4) for 12 h at room temperature.

### 2.2 Experimental description

Three kinds of mechanical tests, namely the uniaxial tensile tests, the uniaxial creep recovery tests and the biaxial creep recovery tests were conducted on the biaxial dynamic mechanical testing system (IPBF-300, CARE Measurement & Control Co., Ltd., China). For uniaxial tensile and uniaxial creep-recovery tests, only one side of the specimen was loaded, leaving the other side of the specimen freely shrinking; for biaxial creep-recovery tests, both loading axes were loaded and recovered at the same time. The uniaxial tensile tests were performed at different strain rates of .01%/s, .1%/s and 1%/s considering the limit of physiological strain rate of articular cartilage (Huang et al., 2003; Li et al., 2003; Li and Herzog, 2004). As reported that the deformation of cartilage under physiological loads is no larger than 30% with the average deformation value lower than 20%. Thus, the rate-dependent tensile tests were stopped when the strain reached 20% (Liu et al., 2010; Wang et al., 2015).

The stress on the cartilage was .535 MPa in double leg stance and .953 MPa in single leg stance (Qiu et al., 2016). In order to approximate the *in vivo* stress state, both uniaxial and biaxial creep-recovery tests were carried out at different stress levels *σ* of .5, 1.0 and 1.5 MPa. In addition, for the biaxial creep-recovery tests, the different biaxial stress ratios (denoted as *B*, indicates the ratio of stress in one loading direction to that in the other direction) of .3, .5 and 1.0 were applied. It can be seen that the biaxial stress ratio *B*) of uniaxial creep tests is 0 and *B* = *1* means that the cartilage is in an equi-biaxial stress state. For all the creep-recovery test conditions mentioned above, the creep time was 2,400 s and followed by the strain recovery (at 0 MPa) time of 3,600 s to make sure that most creep deformation of cartilage has recovered (Eckstein et al., 1999; Erisken et al., 2010).

All the tests were conducted at room temperature with a preload of 0.1 N being applied before tests to keep the samples align with the axis of loading. Besides, to prevent cartilage samples from drying (Erisken et al., 2008), an air humidifier was placed near the sample and a sprayer was employed to spray the PBS solution on the surface of sample every 5 min during tests (Si et al., 2022).

### 2.3 Image and data analysis

**FIGURE 2:**
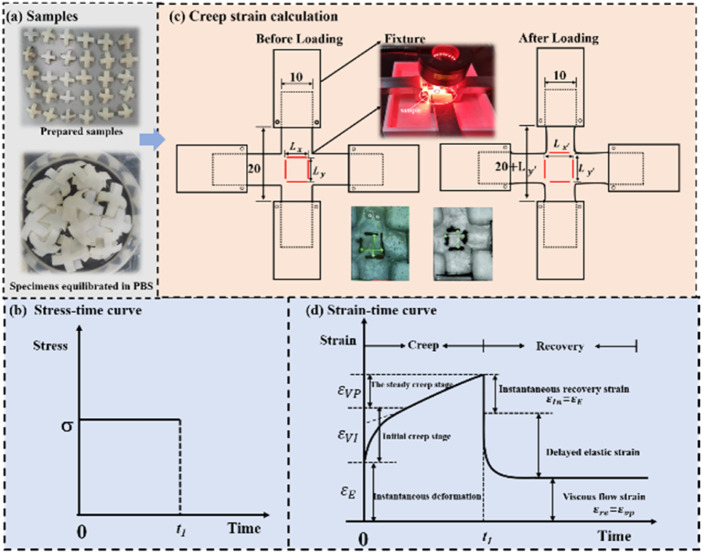
**(A)** Samples; **(B)** stress-time curve; **(C)** creep strain calculation; **(D)** stress-time curve.

During mechanical tests, the load and displacement of the central area (10 mm × 10 mm) were recorded by the mechanical sensor and the digital image correlation (DIC) system equipped by the biaxial dynamic mechanical testing machine. The tensile stress of cartilage is the load divided by the cross-sectional area. The measurement of strain is similar with that reported in literatures (Kamalanathan and Broom, 1993; Lin et al., 2017; Lin et al., 2018; Zhang et al., 2019). As shown in Figure 2C, two pairs of thin lines were drawn in the middle local mark section of cruciform sample. A optical camera was applied to measure the variation of distance between fine lines. The distances between two pairs of marked lines were recorded as length $L_{x\;}$ and $L_{y}$ (before loading) and ${L_{x}}^{\prime }$ ; ${L_{y}}^{\prime }$ (after loading). Thus, the strain in *x*-axis and *y*-axis directions was calculated as:

$$\varepsilon_{x}=\frac{{L_{x}}^{\prime }-L_{x}}{L_{x}},\;\varepsilon_{y}=\frac{{L_{y}}^{\prime }-L_{y}}{L_{y}}$$ (1)

Only the strain in *x*-axis is recorded for uniaxial mechanical tests. For the creep-recovery tests, as shown in Figure 1D, the creep strain is divided into three components, namely instantaneous elastic strain $\varepsilon_{e}$ , delayed elastic strain $\varepsilon_{D}$ and viscous flow strain $\varepsilon_{V}$ .

### 2.4 Statistical analysis

Considering the random error of experiments, three samples were repeated by the same person under the same loading condition, and test data were used in data analysis. The figures were plotted in Origin 2018 and the statistical analyses were performed using SPSS 22.0. R-squared value was used to indicate the agreement between the test result and its prediction. A one-way analysis of variance (ANOVAs) was employed to detect significant differences between different testing conditions. The statistical results were significant if *p* < .05.

## 3 Theoretical model

The viscoelastic constitutive model Eq. 2 introduced by Lou and Schapery is used to describe the unconfined mechanical behavior of cartilage (Lou and Schapery, 1971). The constitutive model under uniaxial loading can be expressed as

$$\varepsilon =g_{0}A\left(0\right)\sigma +g_{1}\overset{t}{\underset{0}{\int }}\Delta A\left(\psi -\psi^{\prime }\right)\frac{dg_{2}\sigma }{d\tau }d\tau$$ (2)

Where $A\left(0\right)$ is initial components of linear viscoelastic creep compliance, and $\Delta A\left(\psi \right)$ is transient components of linear viscoelastic creep compliance. *t* is the loading time. $g_{0}$ , $g_{1}$ and $g_{2}$ are three stress-dependent material parameters and their changes reflect the third and higher order stress-dependence of Gibbs free energy. The converted time ψ is expressed as

$$\psi =\psi \left(t\right)=\overset{t}{\underset{0}{\int }}\frac{dt^{\prime }}{a_{\sigma }},\;\psi^{\prime }=\psi \left(\tau \right)=\overset{\tau }{\underset{0}{\int }}\frac{dt^{\prime }}{a_{\sigma }}$$ (3)

Here $a_{\sigma }$ is the time-scale factor, which reflects the relevance of free energy, stress and temperature. In this study we assume that $a_{\sigma }$ depends only on the applied stress.

When the step-stress input $\sigma \left[H\left(t\right)-H\left(t-t_{1}\right)\right]$ (Figure 2B) is applied, Eq. 2 yields the creep strain

$$\varepsilon_{c}=g_{0}A\left(0\right)\sigma +g_{1}g_{2}\Delta A\left(\frac{t}{a_{\sigma }}\right)\sigma ,\;0<t<t_{1}$$ (4)

And the recovery strain can be expressed as

$$\varepsilon_{r}=g_{2}\left[\Delta A\left(\psi \right)-\Delta A\left(\psi -\psi_{1}\right)\right]\sigma ,\;t>t_{1}$$ (5)

Where in Eq. (5)

$$\psi_{1}=t_{1}/a_{\sigma },\;\psi =t_{1}/a_{\sigma }+t-t_{1}$$ (6)

The creep strain at *t*_*1*_ (just before unloading) can be described as follows in Eq. (7). At the moment after unloading the initial strain in recovery phase can be described as follows in Eq. (8).

$$\varepsilon_{c1}=\varepsilon \left(t_{1}\right)=g_{0}A\left(0\right)\sigma +g_{1}g_{2}\Delta A\left(\psi_{1}\right)\sigma$$ (7)

$$\varepsilon_{r}\left(t_{1}\right)=\varepsilon_{1}^{\prime }=g_{2}\Delta A\left(\psi_{1}\right)\sigma$$ (8)

Therefore, the strain jump value at $t=t_{1}$ can be expressed as

$$\delta \varepsilon_{1}=\varepsilon_{c1}-\varepsilon_{1}^{\prime }=g_{0}A\left(0\right)\sigma +\left(g_{1}-1\right)g_{2}\Delta A\left(\psi_{1}\right)\sigma$$ (9)

The transient component of linear viscoelastic creep compliance is expressed as

$$\Delta A\left(\psi \right)=C\psi^{n}$$ (10)

Where *C* is creep coefficient, *n* is creep index.

By substituting Eq. (10) into Eq. (4), we obtain the non-linear viscoelastic creep model.

$$\varepsilon_{c}=g_{0}A\left(0\right)\sigma +C\frac{g_{1}g_{2}}{a_{\sigma }^{n}}t^{n}\sigma$$ (11)

By substituting Eq. (10) into Eq. (5), we obtain the expression of recovery strain:

$$\varepsilon_{r}=\frac{\Delta \varepsilon_{c}}{g_{1}}\left[{\left(1+a_{\sigma }\lambda \right)}^{n}-{\left(a_{\sigma }\lambda \right)}^{n}\right]$$ (12)

Where in Eq. (12)

$$\lambda =\frac{t-t_{1}}{t_{1}}$$ (13)

and

$$\Delta \varepsilon_{c}=\varepsilon \left(t_{1}\right)-\varepsilon_{0}=g_{1}g_{2}c{\psi_{1}}^{n}\sigma$$ (14)

It is noted that the parameters in creep model Eq. 11 and recovery model (Eqs. 12–14 were obtained by fitting the test data with the non-linear least square method in the software origin 2018. Thus, the uniaxial creep-recovery strains of cartilage can be fitted by the built model.

## 4 Results

### 4.1 Tensile properties of articular cartilage at different strain rates

**FIGURE 3:**
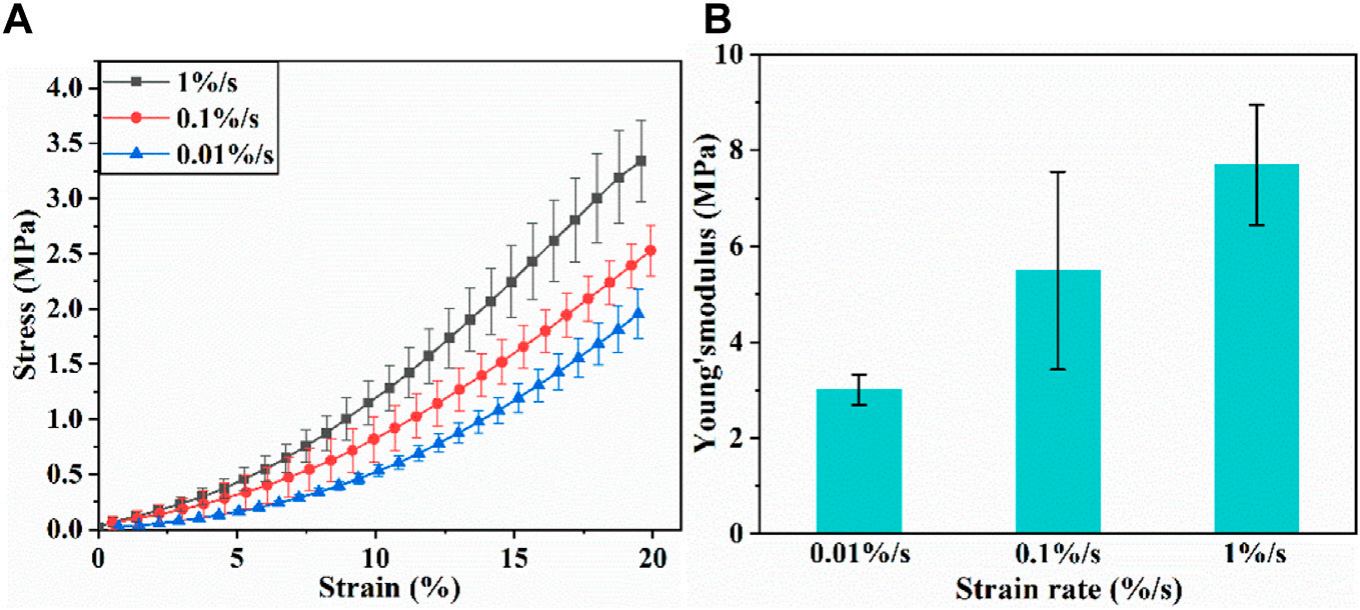
**(A)** Tensile stress-strain curves of articular cartilage; **(B)** Young’s modulus of articular cartilage.

Figure 3A shows the stress-strain curves of articular cartilage at different strain rates. The stress-strain curves are not coincident, indicating that the tensile behavior of articular cartilage is strain rate-dependent. According to literature (Korhonen et al., 2002), and considering the error in data at the very beginning of the experiments, the Young’s modulus of cartilage was determined by calculating the slope of linear range of stress-strain curve when the strain value is 1%–5%. Effect of strain rate on Young’s modulus is shown in Figure 3B. Young’s modulus increases obviously with the increase of strain rate.

### 4.2 Uniaxial creep-recovery behaviors of articular cartilage at different stress levels

**FIGURE 4:**
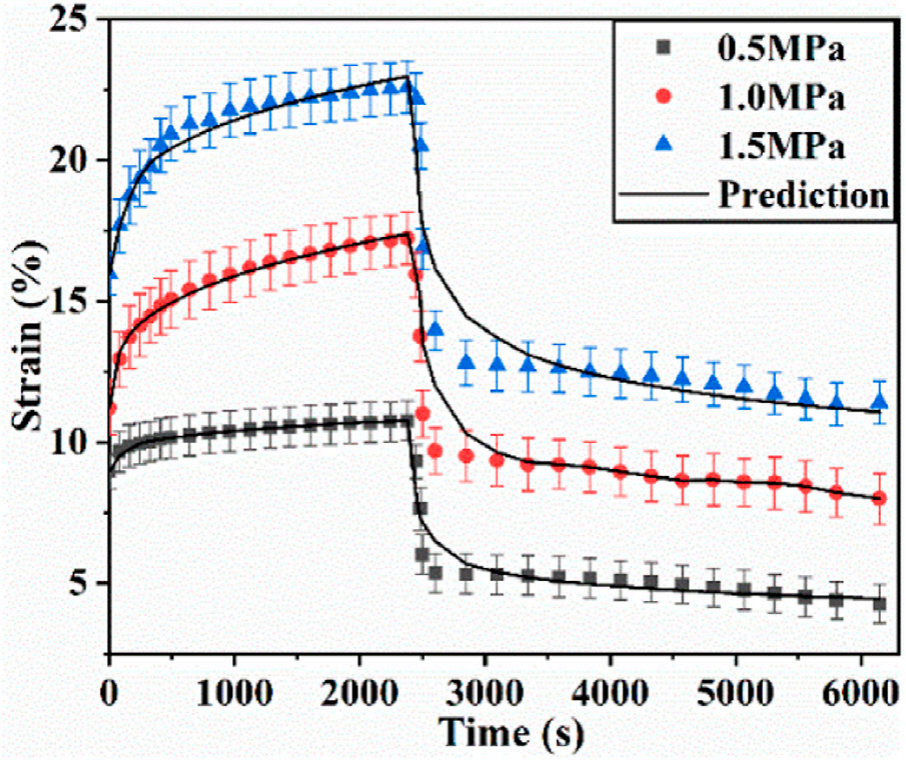
Uniaxial creep-recovery curves of cartilage at different stress levels.

The uniaxial creep-recovery curves of cartilage at different stress levels are shown in Figure 4. In the phase of creep, the creep strain at each stress level increases rapidly at first, and followed by the decrease of growth rate with creep time. The creep strain increases with the increase of stress level (*p* < .05). In the phase of recovery (*σ* = 0 MPa), the strain decreases rapidly at the beginning, and then the declining rate decreases with recovery time. When the recovery time is 2,400 s, the strain decreases to 45.1%, 50.3% and 53.5% of initial recovery strain at stress level of .5, 1.0, 1.5 MPa. When the recovery time is 3,600 s, the recovery strain decreases to 39.5%, 46.4% and 50.5% of initial recovery strain. Enough unloading time is beneficial for creep recovery. And longer recovery time is required for creep recovery at higher stress level.

Simultaneously, the creep strains and recovery strains at different stress levels have also been fitted by the theoretical creep-recovery model (Eq. (11) and Eq, 12. Figure 4 demonstrates the comparisons of test results and fitting results. There are good agreements between them (R > .8).

**FIGURE 5:**
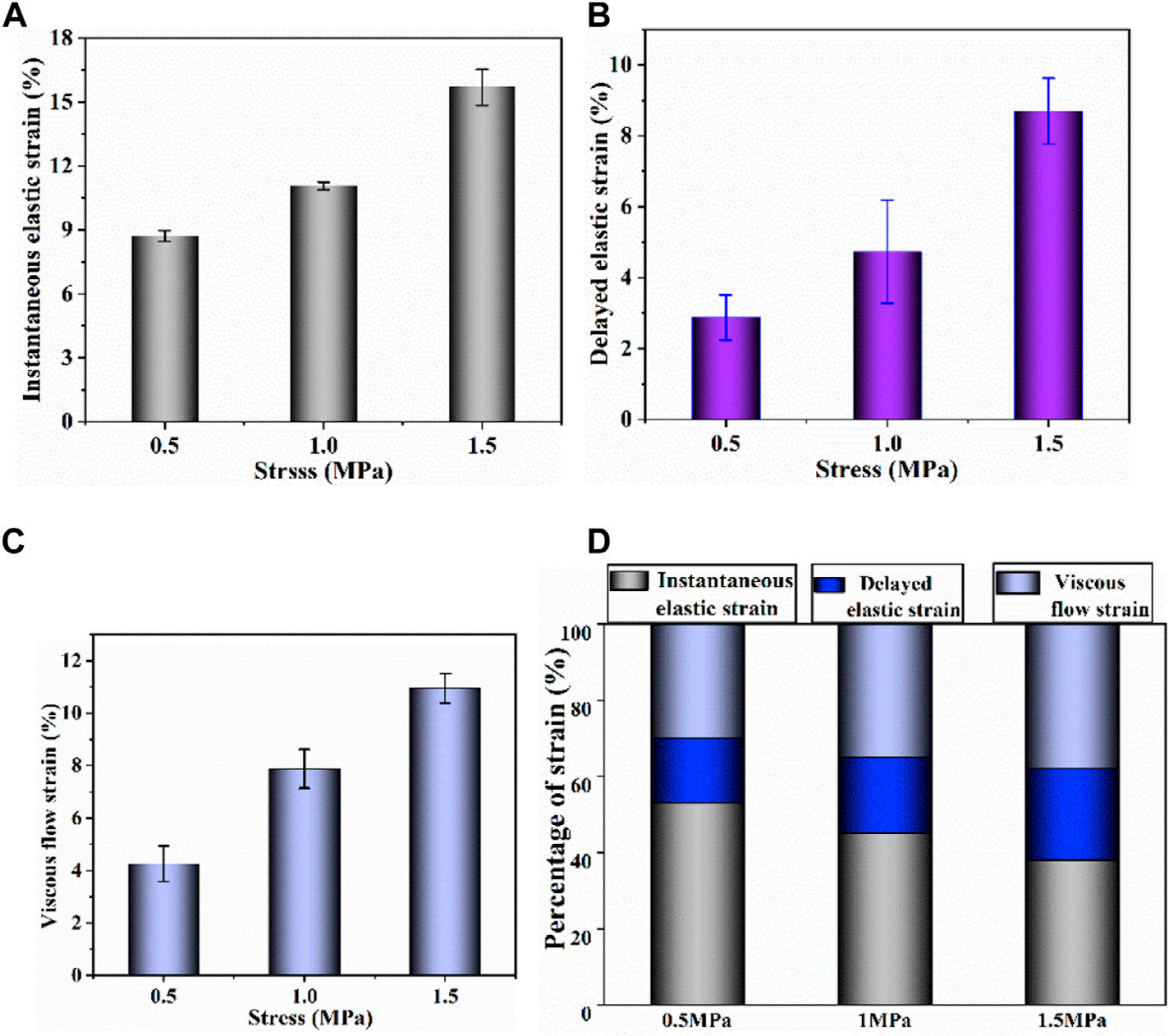
**(A–C)** Instantaneous elastic strain, delayed elastic strain and viscous flow strain components of uniaxial creep strain, respectively; **(D)** percentage of each strain component at different stress.

Based on the creep strain classification in Figure 2D, each component at different stress levels is compared in Figure 5. It is noted that the increase of stress level results in an increase in all strain components. As shown in Figure 5D, by comparing the percentage of each strain component, the contribution of each strain component at different stress levels was evaluated. Decrease in proportion of instantaneous elastic strain and increase in proportions of delayed elastic strain and viscous flow strain are observed with the increase of stress level.

### 4.3 Biaxial creep-recovery behaviors of articular cartilage at different stress levels

**FIGURE 6:**
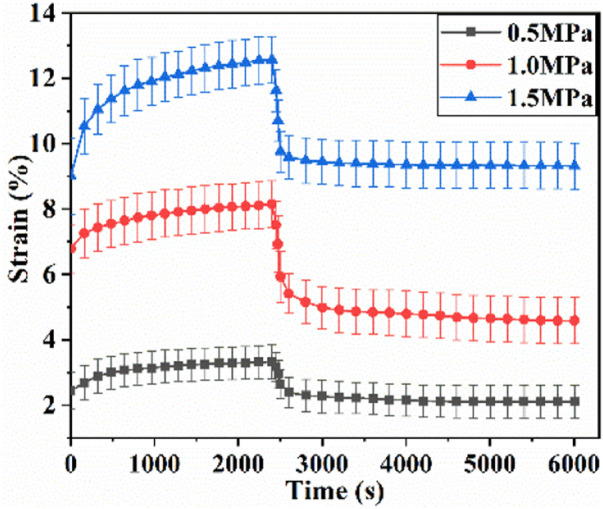
Biaxial creep-recovery curves of articular cartilage at different stress levels.

**FIGURE 7:**
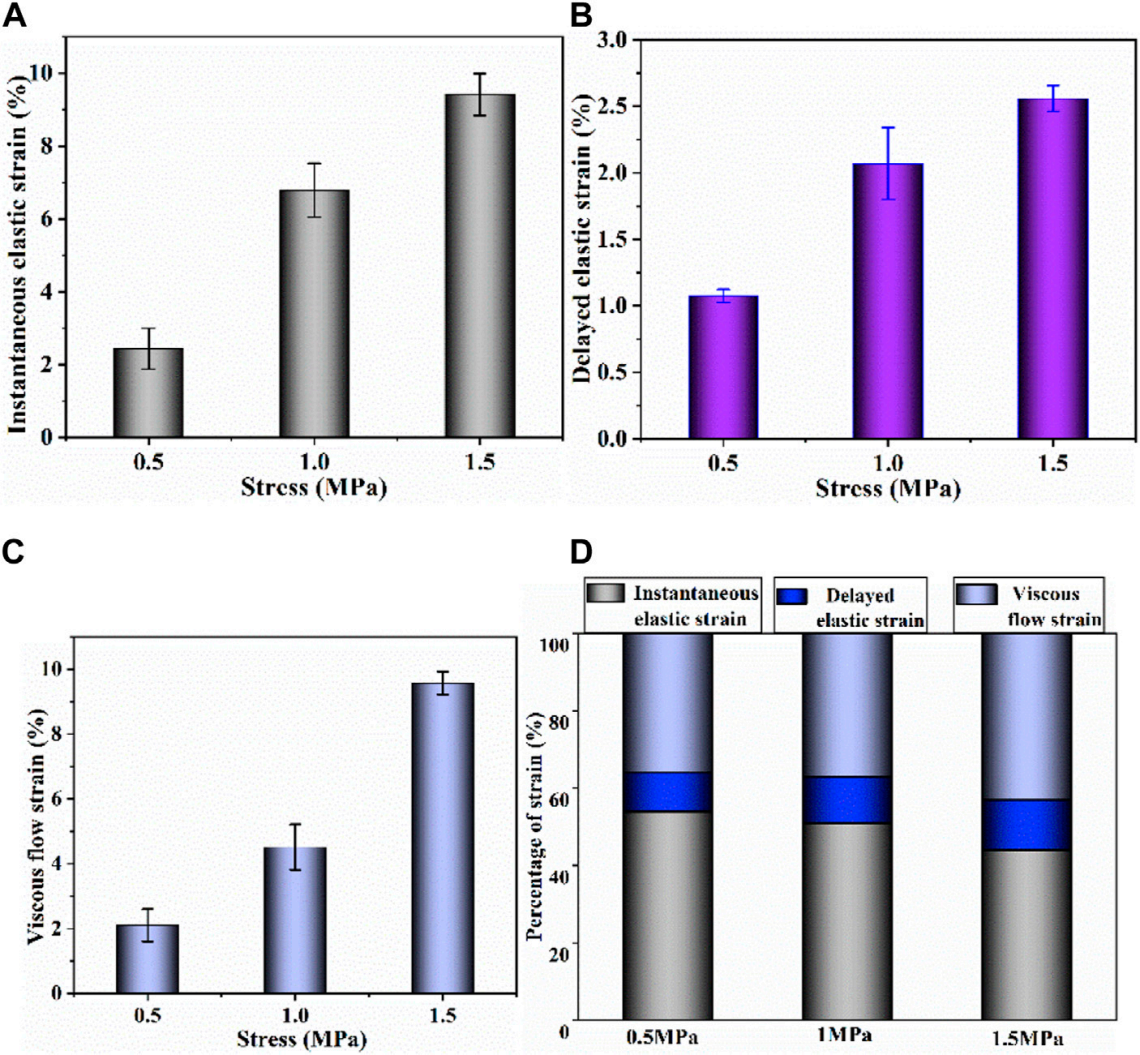
**(A–C)** Instantaneous elastic strain, delayed elastic strain and viscous flow strain components of biaxial creep strain, respectively; **(D)** percentage of each biaxial strain component at different stress levels.

Biaxial creep-recovery tests with different stress levels were carried out at B=1.0 . Only the creep-recovery behavior in *x*-axis (along the split line of cartilage) was chosen for discussion since the creep-recovery responses in two axes are similar. Figure 6 shows the biaxial creep-recovery strain curves at different stress levels. Similar to uniaxial creep response in Figure 4, both biaxial creep strain and the biaxial residual strain increase with the increase of stress level (*p* < .05). The biaxial creep strain is smaller than the uniaxial creep strain, indicating that the equi-biaxial loading enforces constraint on the accumulation of biaxial creep strain. Similarity, the biaxial recovered strain is less than the uniaxial recovered strain at the same stress level, which is illustrated by analyzing the percentages of creep strain components (Figure 7). The recovered strain mainly includes the intantaneous elastic strain and delayed elastic strain (Figure 2D). As shown in Figure 7D, the percentages of biaxial delayed elastic strain with .5, 1.0, 1.5 MPa are 10%, 12%, 13%, which are smaller than those of 17%, 20%, 24% under uniaxial loading. In addition, the percentages of instantaneous elastic strain under biaxial loading are almost as large as those under uniaxial loading. Differences in percentages of these creep strain components suggest that biaxial loading serves to suppress the delayed elastic strain, resulting in the smaller biaxial recovered strain and thus the larger residual strain in biaxial creep-recovery tests.

**FIGURE 8:**
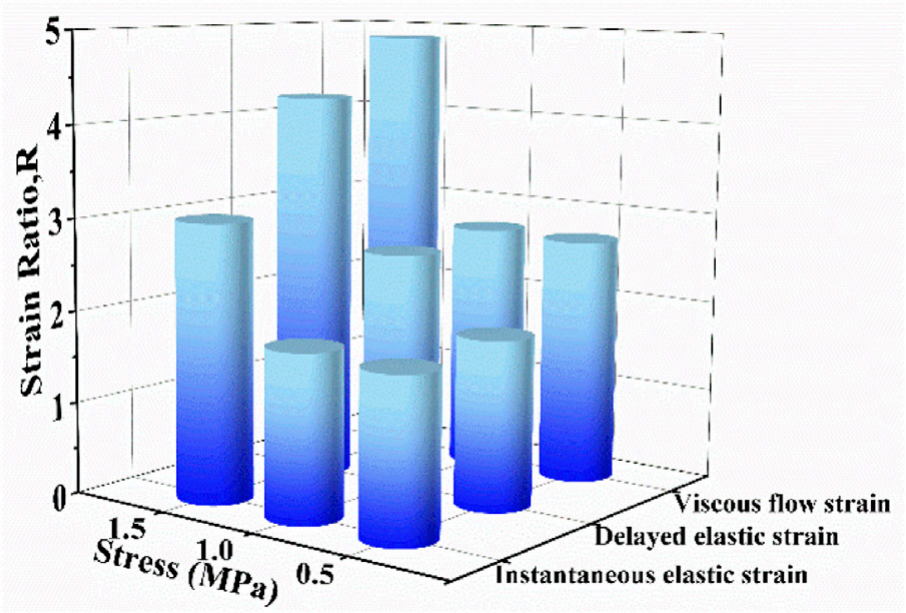
The strain ratio as a function of stress level for instantaneous elastic strain, delayed elastic strain and viscous flow strain.

To further quantify the effect of biaxial stress level on creep strain, the strain ratio *R* was proposed and it is defined as uniaxial creep strain component divided by corresponding biaxial creep strain component. Figure 8 compares the strain ratios of instantaneous elastic strain, delayed elastic strain and viscous flow strain at different stress levels. It is found that all strain ratios at different stress levels are larger than 1, which means that each uniaxial creep strain component is larger than the corresponding biaxial creep strain component. At the same stress level, the strain ratio of viscous flow strain is larger than that of the other two components. The strain ratio increases with the increase of stress level by focusing on certain creep strain component. Compared with .5 MPa and 1.0 MPa, 1.5 MPa exhibits the most remarkable increase in all three creep strain components. Besides, the strain ratios of viscous flow strain and delayed elastic strain experience greater increase than that of instantaneous, regardless the stress level. Consequently, biaxial loading reduces the creep strain by retarding viscous flow strain and delayed elastic strain, especially at high stress of 1.5 MPa.

### 4.4 Effect of biaxial stress ratio on biaxial creep-recovery behavior

**FIGURE 9:**
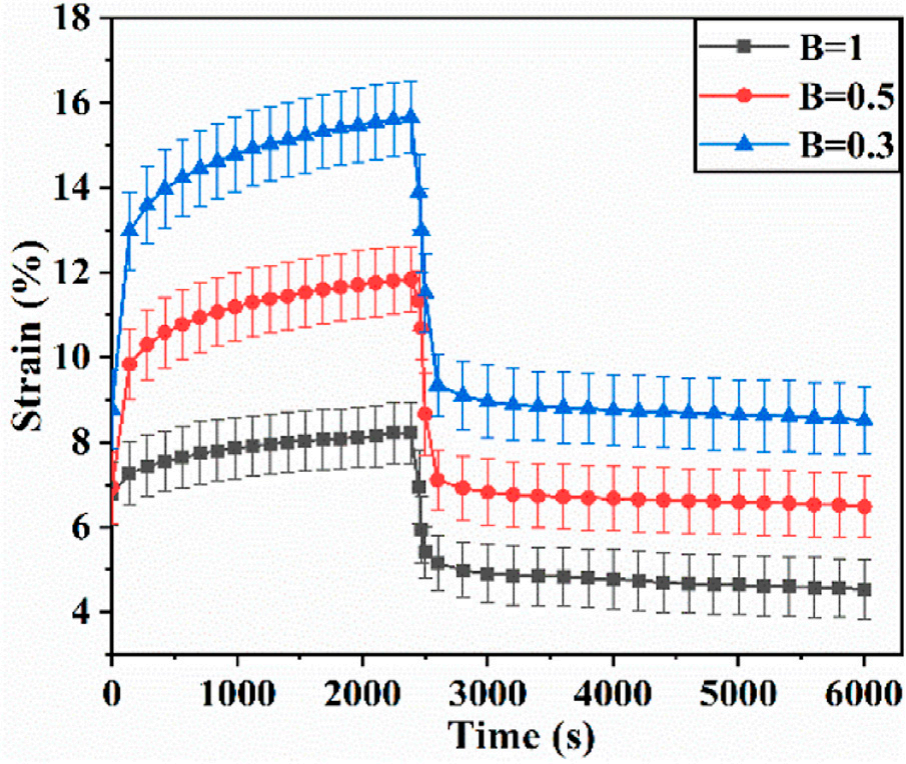
Biaxial creep-recovery curves of articular cartilage at different stress ratio.

**FIGURE 10:**
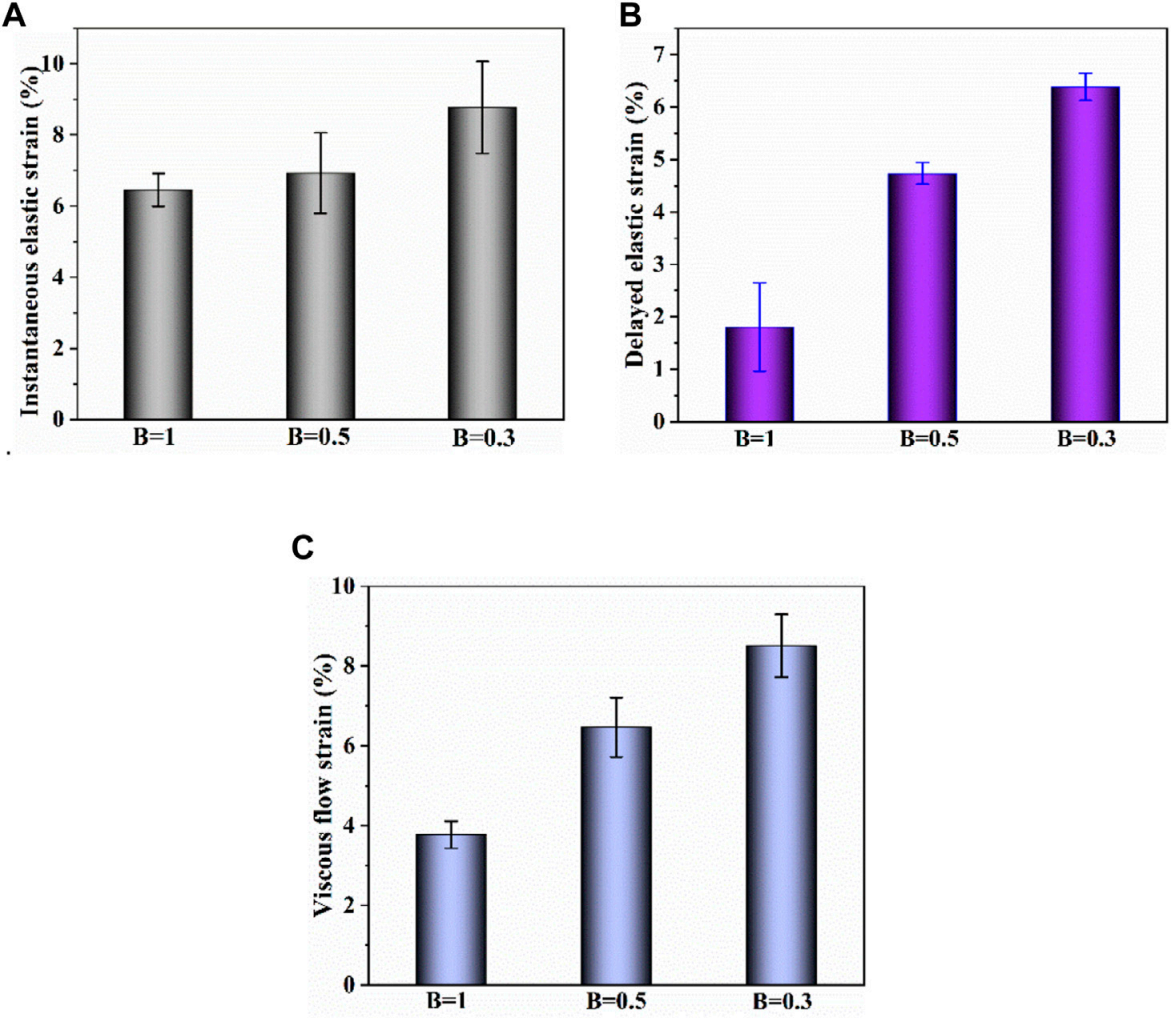
**(A–C)** Instantaneous elastic strain, delayed elastic strain and viscous flow strain components of biaxial creep strain, respectively.

Figure 9 shows the creep-recovery responses of cartilage at different biaxial stress ratios. The biaxial creep strain decreases with increase of biaxial stress ratio (*p* < .05), indicating that the stronger the biaxial constraint, the smaller the creep strain. Besides, the larger biaxial creep strain shows the smaller residual strain. As shown in Figures 10A–C, increasing biaxial stress ratio *B* from .3 to 1.0 brings a large reduction of each creep component, leading to the decreasing of creep and recovered strain. In particular, the delayed elastic strain decreases from 4.74 to 1.81 with biaxial stress ratio from *B* = .5 to *B* = 1.0, corresponding to a 72% reduction.

**FIGURE 11:**
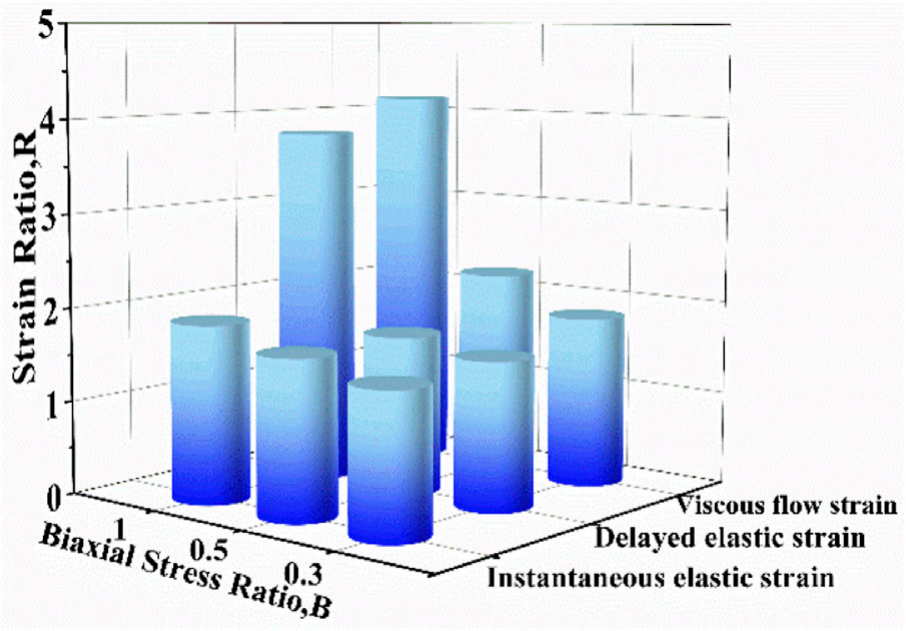
The strain ratio as a function of biaxial stress ratio for instantaneous elastic strain, delayed elastic strain and viscous flow strain.

In addition, the strain ratios of instantaneous elastic strain, delayed elastic strain and viscous flow strain at different stress ratios were compared in Figure 11. At the same stress level, the strain ratio of instantaneous elastic strain is smaller than that of the other two components. The strain ratio of instantaneous elastic strain rises slightly with the rise of biaxial stress ratio. By comparison, the strain ratios of delayed elastic strain and viscous flow strain show the more remarkable increase with the rise of biaxial stress ratio, especially when the biaxial stress ratio increase from *B* = .5 to *B* = 1.0. The results indicate that biaxial loading limits creep deformation of cartilage by inhibiting its delayed elastic strain and viscous flow strain, and the equi-biaxial stress state imposes the largest constriction.

## 5 Discussions

In daily activities, articular cartilage is subjected to long time biaxial loads or multiaxial loads. The creep deformation is induced in cartilage under mechanical loads due to the alternation of unequal fluid exudation and imbibition within its matrix. Once unloaded, most of the creep deformation will gradually recover with time. Uniaxial creep-recovery of cartilage have been well described in previous studies (Gao et al., 2019; Cutcliffe and DeFrate, 2020). However, seldom has any study focused on biaxial or multiaxial creep-recovery of cartilage. Thus, we probed the creep-recovery behaviors of articular cartilage by comparing uniaxial and biaxial tensile loading results.

The creep strain evolution of cartilage consists of two parts, namely initial creep stage and the steady creep stage. In initial creep stage, the uniaxial and biaxial creep strain showed a similar evolution that the creep strain increased rapidly at the beginning, and gradually tended to be stable with time (Figure 4; Figure 6; Figure 9). The rapid increase of strain in initial creep stage for cartilage is due to the rapid outflow of interstitial fluid. When the unbound fluid in cartilage is squeezed out, the burden of supporting tensile load is gradually shifted to the collagen network. The cartilage becomes more resistant to deformation and the next creep stage starts. In steady creep stage, the increase rate of creep strain stays constant. The creep strain evolution observed in this study is similar with that reported by Hosseini et al. and Choi et al. as they found that the creep deformation *in vivo* kept stable after a rapid growth (Hosseini et al., 2010; Choi et al., 2016). However, it should be mentioned that there is no accurate cut-off point between the first creep stage and the steady creep stage since the creep deformation of cartilage varies with different physiological loads (Schoenbauer et al., 2015; Wang et al., 2015; Chan et al., 2016; Choi et al., 2016). Compared with uniaxial creep, biaxial creep exhibits smaller strain at the same stress level, especially at high stress level of 1.5 MPa. It is found that biaxial loading reduces the creep strain through retarding all creep strain components (Figure 5; Figure 7), especially through retarding delayed elastic strain and viscous flow strain (Figure 8). The smaller strain under biaxial creep is ascribed to the higher constraint imposed by the equi-biaxial stress state, as higher constraints can increase the stiffness of the cartilage and restrict the rapid outflow of interstitial fluid.

Generally, the deformation of articular cartilage can be fully recovered within a sufficient period of unloading time due to the viscoelastic properties of articular cartilage (Torzilli, 1984; Athanasiou et al., 1994). Eckstein et al**.** suggested at least 90 min of rest might be required to allow articular cartilage to fully recover according to the imaging based studies (Eckstein et al., 1999). Erisken et al. reported that 1,000 s was sufficient for the compressive stress to relax in bovine cartilage (Erisken et al., 2010). In this study, it is found that both uniaxial and biaxial strain have not been fully recovered with the residual strains left when the recovery time is 3,600 s. Compared with uniaxial loading, biaxial loading shows a smaller residual strain (Figure 4; Figure 6; Figure 9), due to the significant decrease in the viscous flow strain at equi-biaxial stress state (Figure 5C; Figure 7C). The residual strain, as viscous flow strain, is slow to recover. Thus, more time may be needed for the residual creep deformation to recover.

It is very likely for cartilage to experience non-uniform stress distribution in daily activities due to its curvature structure, thickness variation, diffusion kinetics and other factors (Kamalanathan and Broom, 1993). Kamalanathan and Broom reported that the tissue was significantly stiffer, i.e. more strain limiting, along the split-line direction than across it. They found that the tissue was significantly stiffer, i.e. more strain limiting, along the split-line direction than across it and the anisotropic strain-limiting response of the fibrillar array will result in fibrils acting more collectively along the less extensible direction than across it. Obviously, the stress state of cartilage under physiological load is not equi-biaxial. To better understand how biaxial constraint influences biaxial creep behavior, we studied the effect of biaxial stress ratio on creep-recovery behavior of cartilage (Figure 8). Both creep strain and recovered strain decrease with the increase of biaxial stress ratio due to the reason that the larger the biaxial stress ratio, the stronger the biaxial constraint. The equi-biaxial stress state (*B* = 1) imposes the largest biaxial constraint. Thus, when the biaxial stress ratio *B* is 1.0, the lowest creep and recovery strain is induced in cartilage. Increasing the biaxial stress ratio from .3 to 1.0 causes a decrease in all creep strain components, especially in delayed elastic strain (Figure 9; Figure 10). Furthermore, less proportion of delayed elastic strain results in less time to reach steady recovery state (Figure 8) since delayed elastic strain is slow to recover. In addition, the higher constraint was imposed by the larger biaxial stress ratio, which in turn increases the stiffness of cartilage (Lin et al., 2017; Zhang et al., 2019) resulting in the smaller creep strain and residual strain.

Viscoelastic (Leipzig and Athanasiou, 2005; Si et al., 2022), biphasic (Huang et al., 2003) and poroviscoelastic models (Wang et al., 2001) are employed to describe mechanical behaviors of cartilage in literature. Leipzig and Athanasiou used the viscoelastic model to describe the mechanical behaviors of cartilage cells (Leipzig and Athanasiou, 2005). Gao et al. employed the non-linear viscoelastic constitutive model to predict the depth-dependent creep behaviors of cartilage under compression (Gao et al., 2014). The viscoelastic constitutive model employed in this study is proposed by Lou and sharper (Lou and Schapery, 1971). It has the outstanding advantage of retaining the single time integral form even in the non-linear range. And the non-linear effects are expressed by means of stress-dependent material functions determined experimentally. It has been applied to predict the mechanical behaviors of cracked cartilage and exhibits good results (Si et al., 2022). In this study, the proposed viscoelastic model with the consideration of irrecoverable deformation was developed to describe the uniaxial creep-recovery behaviors of cartilage and a good consistence between test data and fitted data is obtained.

## 6 Conclusion

The study probed the uniaxial and biaxial creep-recovery behaviors of articular cartilage. Changing the stress state from uniaxial loading to biaxial loading, creep strain decreases significantly under the same stress level. Increasing biaxial stress ratio (from *B* = .3–1) causes notable decrease in creep strain. During the recovery process of creep, it takes less time for the biaxial strain than the uniaxial strain to reach the steady recovery state. The cartilage with higher stress level or smaller biaxial stress ratio shows larger residual strain, indicating that the cartilage damage may be accelerated at higher stress level or at smaller biaxial stress ratio. These results provide a new sight for cartilage creep and are significant to improve the durability of cartilage in daily activities.

## Data Availability

The original contributions presented in the study are included in the article/supplementary material, further inquiries can be directed to the corresponding authors.
